# Complete Genome Sequence of Burkholderia contaminans SK875, Isolated from the Respiratory Tract of a Pig in the Republic of Korea

**DOI:** 10.1128/MRA.00642-20

**Published:** 2020-07-02

**Authors:** Hae-In Jung, Sang-Won Lee, Soo-Ki Kim

**Affiliations:** a Department of Animal Sciences and Technology, Konkuk University, Seoul, Republic of Korea; b College of Veterinary Medicine, Konkuk University, Seoul, Republic of Korea; Indiana University, Bloomington

## Abstract

Burkholderia contaminans SK875 was isolated from the respiratory tract of a pig in the Republic of Korea. Here, we report the genome of *B. contaminans* SK875, which consists of three circular chromosomes and one plasmid of 8,596,045 bp with 7,727 genes.

## ANNOUNCEMENT

In the past few decades, the Burkholderia cepacia complex (Bcc) has been increasingly reported as secondary or opportunistic pathogens that cause respiratory diseases in humans and animals when ingested or inhaled (1–4). B. contaminans was reported to occur as respiratory tract colonization rather than immediate infection, and some chronic patients with cystic fibrosis develop opportunistic infection (5, 6).

In this study, we provide a detailed description of the complete genomic sequence of *B. contaminans* SK875, which was originally isolated from the respiratory tract of a pig in the Republic of Korea (7). *B. contaminans* SK875 was grown aerobically at 37°C in modified LB (lysogeny broth/Luria Bertani) (7). The genomic DNA of *B. contaminans* SK875 was extracted using the Wizard genomic DNA purification kit (Promega Corporation, Madison, WI, USA) according to the manufacturer’s instructions. The genome of *B. contaminans* SK875 was completely sequenced using the Illumina HiSeq 2000 platform and the PacBio RS II system. The Illumina HiSeq 2000 libraries were prepared using the TruSeq DNA PCR-free kit, and the PacBio RS II system libraries were prepared using the SMRTbell template prep kit v1.0. In total, 129,245 PacBio subreads with 1,077,431,117 bp were generated using the PacBio RS II system, and their mean length and *N*_50_ value were 8,336 and 11,095 bp, respectively. The sequencing reads were *de novo* assembled using HGAP3 with default options, yielding four contigs harboring 8,596,045 bp (8, 9). The assembly was completed with the PacBio RS II system. The Illumina HiSeq 2000 libraries were used for error correction. The sequence quality was checked using FastQC, and the reads were filtered before assembly to keep only paired-end (PE) reads having more than 90% of bases with a base quality greater than or equal to Q20. Error correction of the tentatively complete circular sequences was performed using the default option of five replicates in ICORN2. As a result, the whole-genome sequencing and *de novo* assembly produced four contigs; the ends of the contigs overlapped and connected to each other to form a circle, matching the sequences of three chromosomes and one plasmid.

Annotation of the open reading frames and functional gene analysis were carried out using Prokka v1.10 (10). The general characteristics of the whole-genome sequence of *B. contaminans* SK875 were analyzed using Geneious v8.1.9 software (11). The complete genome of SK875 was comprised of three circular chromosomes (3,618,903 bp, 3,247,714 bp, and 1,528,467 bp) and one plasmid (200,961 bp), with respective GC contents of 66.4%, 66.7%, 65.8%, and 61.7% (Table 1). A total of 7,727 genes were identified from the three chromosomes and the plasmid, with 3,246, 2,841, 1,307, and 227 coding DNA sequences (CDSs), respectively. Totals of 18 rRNAs, 83 transfer RNAs (tRNAs), and 1 transfer-messenger RNA (tmRNA) locus, as well as 3 CRISPR elements, 16 prophage regions, and 16 insertion elements (IS), were identified. A multireplicon genome structure is found in most *Burkholderia* species with various numbers of chromosomes and plasmids (12). The large size and repartition of the genomes of *Burkholderia* species are thought to increase their capability to acquire and lose genes (4).

**TABLE 1:** General characteristics of the Burkholderia contaminans SK875 whole genome

Property	Data for:			
	Chromosome 1	Chromosome 2	Chromosome 3	Plasmid
Length of sequence (bp)	3,618,903	3,247,714	1,528,467	200,961
GC content (%)	66.4	66.7	65.8	61.7
No. of open reading frames	3,246	2,841	1,311	227
No. of genes	3,323	2,856	1,320	228
No. of noncoding regions	471,846	440,467	219,597	32,676
% coding	87	86	86	84
No. of RNAs	77	15	9	0
No. of rRNAs	12	3	3	0
No. of tRNAs	64	12	6	1
No. of tmRNAs	1	0	0	0
No. of CRISPR regions	1	2	0	0
No. of prophages	11	4	0	1
No. of IS	4	3	4	5

In conclusion, we present the complete genome of *B. contaminans* SK875. The results provide important information for evaluating this strain’s potential as an opportunistic pathogen. Furthermore, the high-quality complete genome sequences generated for this study will provide valuable information for a deeper understanding of the molecular mechanisms of *Burkholderia* species pathogenesis in future studies.

### Data availability.

These data have been deposited in DDBJ/ENA/GenBank under BioProject accession number PRJNA439184 and SRA accession numbers SRR10321932 and SRR10321933.
